# Mild metatropic dysplasia: emphasis on the magnetic resonance imaging of articular cartilage thickening

**DOI:** 10.1259/bjrcr.20200155

**Published:** 2020-11-04

**Authors:** Fumiko Hamabe, Hiromi Edo, Taro Yamashita, Hiroshi Matsumoto, Soichiro Tamada, Koji Sumi, Hiroshi Shinmoto

**Affiliations:** 1 Department of Radiology, National Defense Medical College, Saitama, Japan; 2 Department of Orthopedic Surgery, National Defense Medical College, Saitama, Japan; 3 Department of Pediatrics, National Defense Medical College, Saitama, Japan

## Abstract

Metatropic dysplasia (MD) is a rare skeletal disorder characterized by short stature due to epiphyseal cartilage and growth plate abnormalities. The severity of MD varies from mild to lethal. This disorder is caused by mutations in the *transient receptor potential vanilloid 4 (TRPV4*) that encodes calcium-permeable, nonselective cation channels.

A 33-year-old female presented at our hospital with a history of worsening knee pain diagnosed at the previous institution as a case of osteoarthritis. Radiographs of the knee showed epiphyseal irregularity without joint space narrowing. On MRI, fat-suppressed proton density-weighted imaging revealed thickened articular cartilage with a smooth surface and an abnormal signal intensity of the subchondral bone; *T*_1_ weighted imaging demonstrated irregularity of the epiphysis. These findings and the familial history (both her children had *TRPV4* mutations) led to the suspicion that her condition could be due to mosaicism for *TRPV4* mutation. To the best of our knowledge, this is the first report of MRI findings focusing on articular cartilage thickening in a patient with mild MD. Bone dysplasia including MD should be considered in young patients with articular cartilage thickening and subchondral bone irregularities on MRI.

## Introduction

Metatropic dysplasia (MD) was first described by Maroteaux *et al* in 1966.^1^ It is a rare form of skeletal dysplasia previously called as “hyperplastic achondroplasia.” In recent times, it has been recognized as a clinical disease with a spectrum ranging from non-lethal to lethal forms.^2,3^ It is an autosomal dominant disorder caused by *transient receptor potential vanilloid 4 (TRPV4*) mutations.^2,4^ While multiple radiographic images of typical MD are widely available, there is no published literature concerning mild MD imaging findings, particularly those of MRI. Here, we report, for the first time, the MRI findings in a mild MD patient focusing on the articular cartilage of the knee joint. MRI may potentially aid in the identification of milder skeletal disorder cases.

## Clinical presentation

A 33-year-old female experiencing bilateral knee pain since the age of 20 was diagnosed with osteoarthritis by the previous hospital. She presented to our hospital (National Defense Medical College Hospital) because of worsening of the knee pain, with greater pain in the left side than in the right. Her height was within the normal range (158.8 cm).

## Investigations/Imaging findings

**Figure 1.**
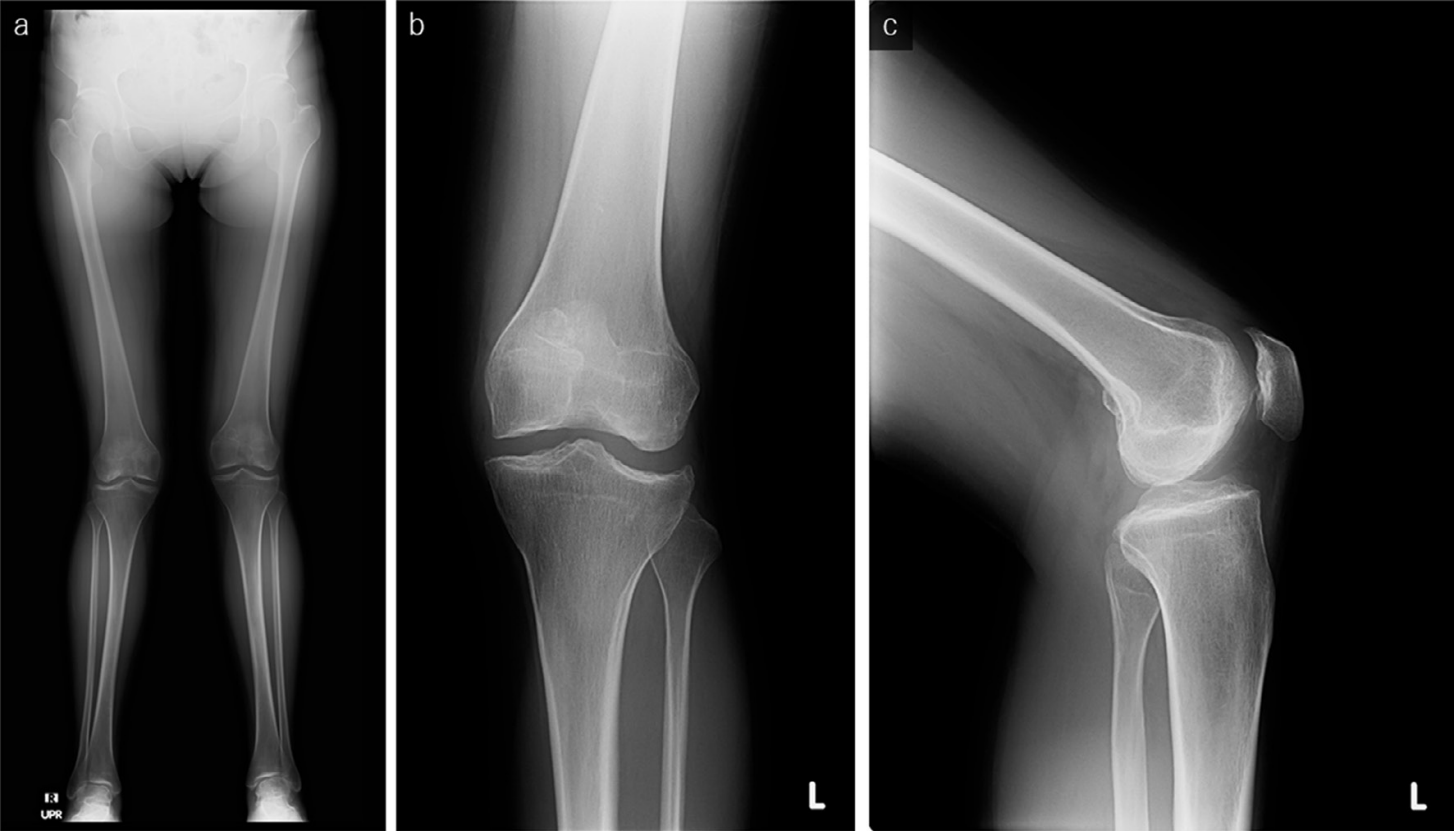
The full-length standing radiograph (a) shows genu valgum. Anteroposterior (b) and lateral (c) radiographs of the left knee show the irregularity of the subchondral bone without joint space narrowing and remarkable osteophytes.

**Figure 2.**
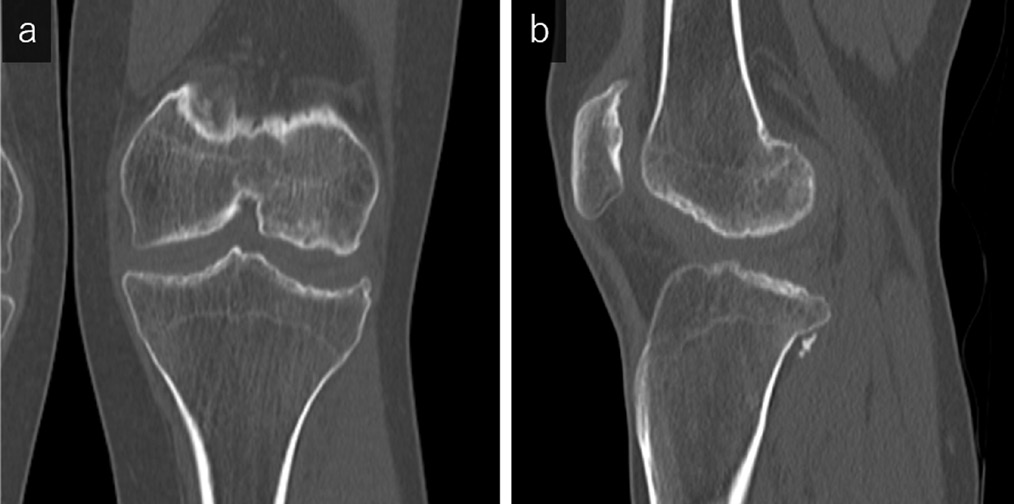
The coronal (a) and sagittal (b) bone window CT scans show the preservation of the joint space despite the subchondral bone sclerosis and irregularities.

A full-length standing radiograph showed genu valgum (Figure 1a). Anteroposterior radiography of the left knee demonstrated epiphyseal irregularities without joint space narrowing (Figure 1b and c). CT images of the knee also clearly depicted the preservation of the joint space despite the subchondral bone sclerosis and irregularities (Figure 2).

**Figure 3.**
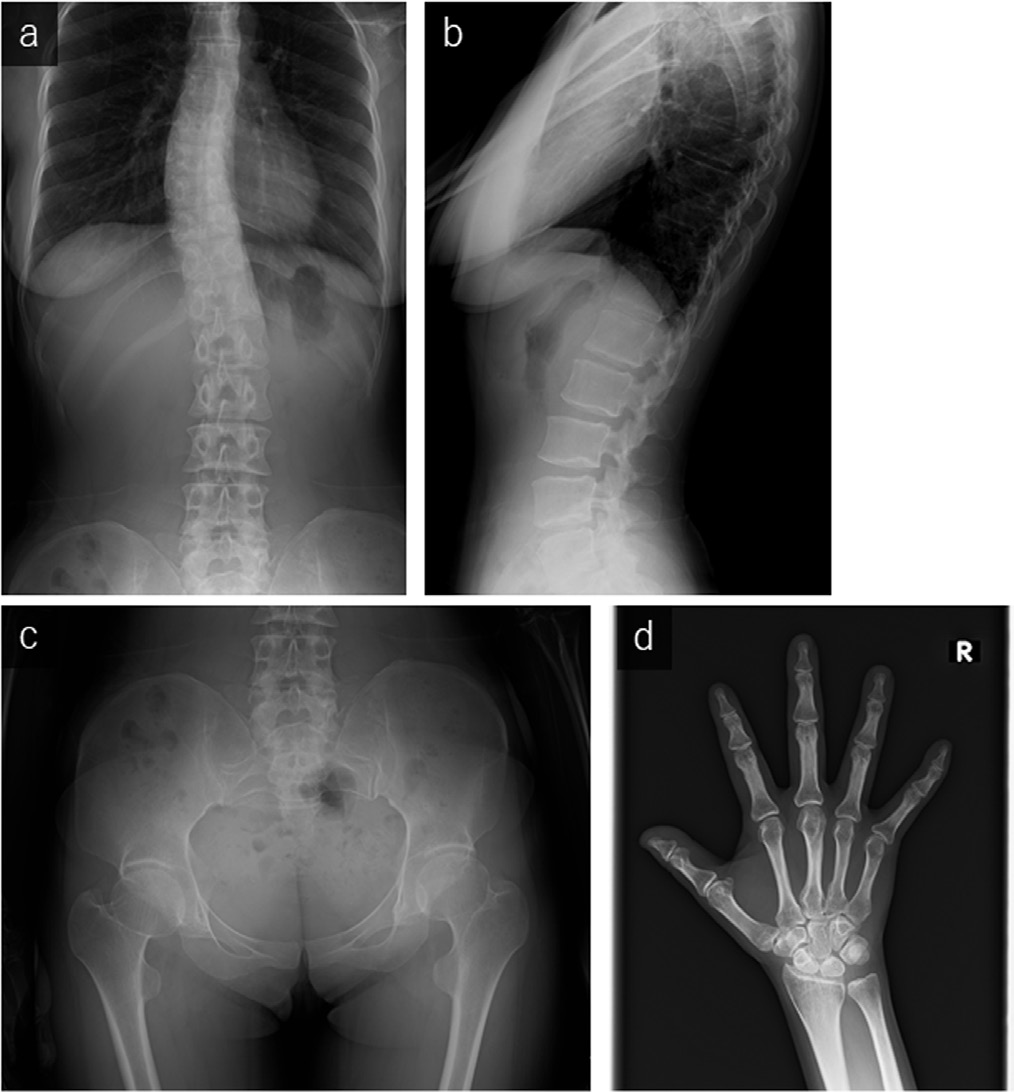
Radiographs of the present case. Anteroposterior (a) and lateral (b) radiographs of the spine demonstrate mild scoliosis without platyspondyly. The anteroposterior radiograph of the pelvis (c) and posteroanterior radiograph of the right hand (d) show no obvious abnormal findings.

**Figure 4.**
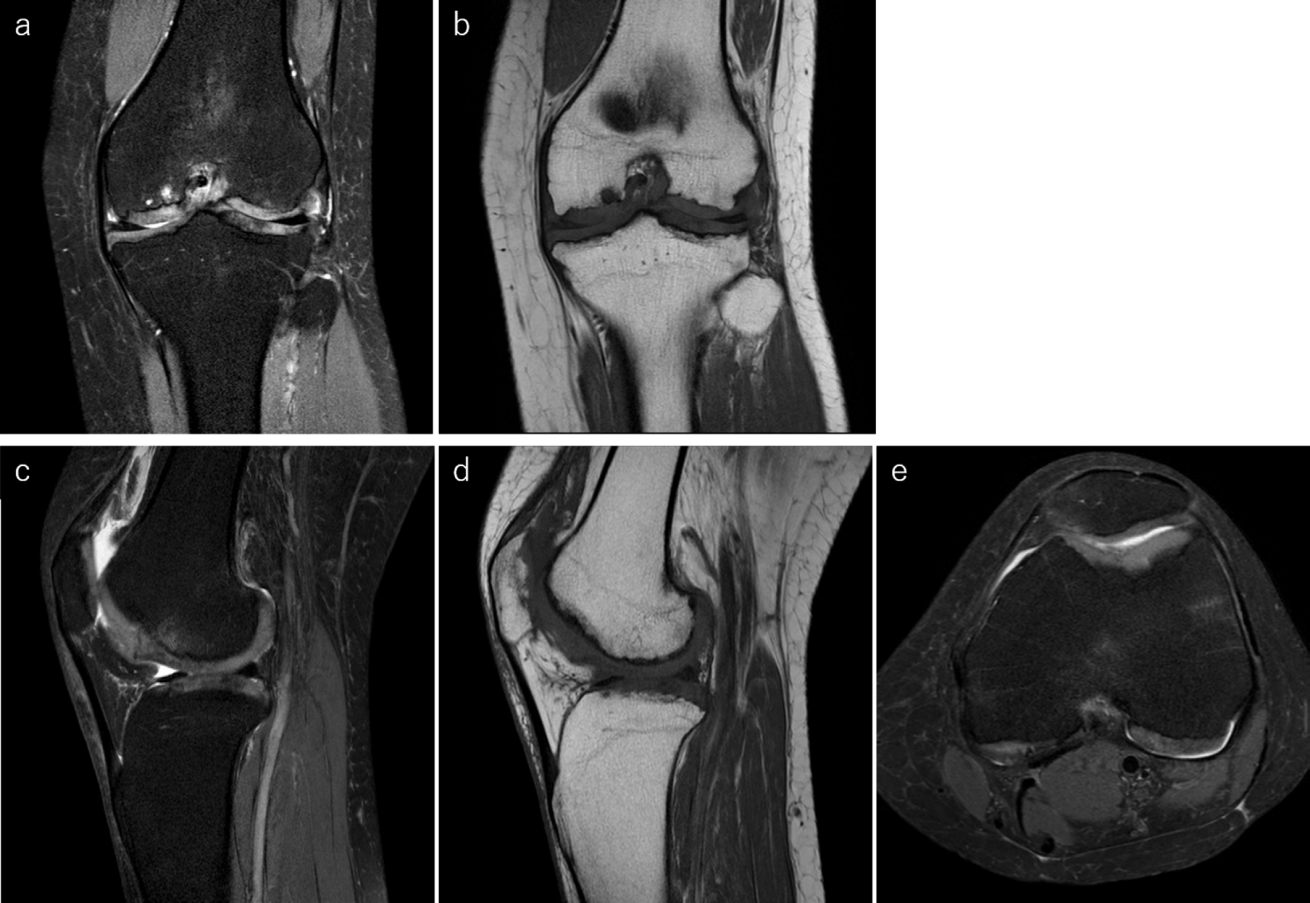
On MRI, coronal (a) and sagittal (c) fat-suppressed proton density-weighted images show a thickened articular cartilage with a smooth surface, irregularity between the subchondral bone and the articular cartilage, and high signal intensity of the subchondral bone. Coronal (b) and sagittal (d) *T*_1_ weighted images show the irregularity of the subchondral bone more clearly. Axial fat-suppressed proton density-weighted image (e) shows thick articular cartilage in the patellofemoral joint as well. There is no evidence of loose body and meniscus damage.

No abnormalities were detected in other joints suggestive of MD (Figure 3). On MRI, fat-suppressed proton density-weighted imaging revealed thickened articular cartilage with a smooth surface and an abnormal signal intensity of the subchondral bone (Figure 4a, c and e); *T*_1_ weighted imaging demonstrated irregularity of the epiphysis (Figure 4b and d).

Familial history revealed that both of her children had been diagnosed with MD, following which her family underwent genetic analysis. Both the children harbored a *TRPV4* mutation (c.2396*C* > T p.Pro799Leu), which is responsible for MD, and her electropherogram was highly suggestive of mosaicism of *TRPV4* mutation. The patient’s spouse did not have the mutation in question.

## Differential diagnosis

In this case, MRI examination demonstrated characteristic findings such as the thickening of articular cartilage with an irregularity of the subchondral bone. These findings on MRI were completely different from those seen with osteoarthritis, and similar findings on MRI in previous reports have been documented as “multiple epiphyseal dysplasia.”^5–7^ Pathologically, it is known that in hyperchondrogenesis, disordered chondrocyte maturation and irregular endochondral ossification occur in varying degrees.^2,3^ These pathological features were consistent with the MRI findings in this case. Among skeletal dysplasia, multiple epiphyseal dysplasia is more frequent than MD, and it is supposed that impaired endochondral ossification causes the delayed ossification of epiphyses during bone growth, thereby resulting in epiphyseal cartilage thickening.^5^ With the thickening of the articular cartilage and the presence of irregularity of the subchondral bone at a young age with knee pain, we suspected MD considering the positive familial history for the mutation. However, if this occurs sporadically, the differential diagnosis should include more frequent multiple epiphyseal dysplasia.

## Discussion

Apart from MD, a rare skeletal dysplasia, *TRPV4* mutations show a variety of phenotypes, and the relationship between the genotype and phenotype is not entirely clear.^3^ Classic MD can be recognized at birth by short limbs with a rapidly progressive spinal deformity in the later part of life which results in a short trunk.^3^ The following two mechanisms may be responsible for the characteristic skeletal abnormalities in MD such as the dumbbell deformity of the long bones and platyspondyly^3,8^: (1) overgrowth of the cartilage in the perichondral ring, and (2) dysfunctional endochondral ossification. In addition, one previous report had proposed mild cases without characteristic skeletal abnormalities.^2^

The *TRPV4* gene encodes calcium-permeable, nonselective cation channels and is broadly expressed in many cell types and tissues such as skin, neurons, bone, and cartilage and can be activated through various stimuli, including temperature, acidic pH, and mechanical loading.^2,3,9^ Activation of *TRPV4* was shown to promote chondrogenesis^10^; which may cause hyperplasia of the epiphyseal cartilage and growth plates through an abnormal reaction to mechanical loading.^8^ On the contrary, the interstitial longitudinal growth of the cartilage was significantly impaired. The imbalance between the perichondral ring and endochondral ossification could be responsible for the characteristic skeletal dysplasia in MD.^3^ The MRI findings in our patient’s knee indicated thick articular cartilage with a smooth surface, and the irregularity between the subchondral bone and articular cartilage could have been due to *TRPV4* mutations. Recent investigations have discovered multiple genetic defects in over 400 different genes in the field of skeletal disorders,^4^ and it is impractical to investigate all of them comprehensively if a skeletal abnormality is found in a patient. Spranger summarized the diseases that had common findings in radiographs as a “family” and suggested that morphological similarities may imply etiological similarities.^11^ Therefore, to narrow down the gene to be investigated, knowing the location of skeletal abnormality on radiographic imaging and the group it belongs to becomes a prerequisite. “Clinical diagnosis” and “radiographic diagnosis” are important to direct and aid in the genetic diagnosis. Keeping the present case in mind, we believe that some patients who were diagnosed with osteoarthritis of the knee based on the radiographs and clinical symptoms may have skeletal dysplasia such as mild MD, as in this case. Radiologists and orthopedic surgeons should read radiographs with utmost care when osteoarthritis of the knee is suspected, especially in young patients. If the thickening of the articular cartilage is suggested on the radiographs, such as preservation of joint space despite the subchondral bone irregularities, MRI can be useful. Potential skeletal abnormalities can be detected on MRI, providing an opportunity to evaluate skeletal dysplasia and helping in revealing genetic variants. Since skeletal dysplasias are hereditary, careful examination is warranted, and it is invaluable to search for the genes responsible, including the possibility of inheritance. As MD is rare, the details of the pathogenesis of the disorders caused by *TRPV4* mutations are not yet known, and further studies are needed for confirmation of these findings.

In conclusion, we report the characteristic MRI findings of articular cartilage thickening in a case of mild MD. Prompt evaluation of the articular cartilage using MRI in suspected cases may lead to early detection of skeletal dysplasias such as mild MD or multiple epiphyseal dysplasia.

## Learning points

1. A thickening of the articular cartilage on MRI may indicate a mild skeletal dysplasia.
2. Evaluation of articular cartilage status by MRI is recommended in young patients with a diagnosis of osteoarthritis of the knee based on radiographs and clinical symptoms and if thickening of the articular cartilage is suggested on radiographs, such as preservation of joint space despite the subchondral bone irregularities.
